# Identification of a coagulation‐related signature correlated with immune infiltration and their prognostic implications in lung adenocarcinoma

**DOI:** 10.1111/1759-7714.15121

**Published:** 2023-10-05

**Authors:** Siqian Yang, Shiqi Chen, Yue Zhao, Tao Wu, Yuquan Wang, Tingting Li, Liwan Fu, Ting Ye, Yue‐Qing Hu, Haiquan Chen

**Affiliations:** ^1^ Department of Thoracic Surgery and State Key Laboratory of Genetic Engineering Fudan University Shanghai Cancer Center Shanghai China; ^2^ Institute of Biostatistics, School of Life Sciences, Human Phenome Institute Fudan University Shanghai China; ^3^ Institute of Thoracic Oncology Fudan University Shanghai China; ^4^ Department of Oncology Shanghai Medical College, Fudan University Shanghai China; ^5^ Sheng Yushou Center of Cell Biology and Immunology, Joint International Research Laboratory of Metabolic & Developmental Sciences, School of Life Science and Biotechnology Shanghai Jiao Tong University Shanghai China; ^6^ Center for Non‐communicable Disease Management Beijing Children's Hospital Beijing China; ^7^ Shanghai Center for Mathematical Sciences Fudan University Shanghai China

**Keywords:** coagulation‐related genes, immune infiltration, immunotherapy, lung adenocarcinoma, prognosis

## Abstract

**Background:**

Lung adenocarcinoma (LUAD) is a fatal form of lung cancer with a poor prognosis. Coagulation system had been confirmed closely related to tumor progression and the hypercoagulable state encouraged the immune infiltration and development of tumor cells, leading to a poor prognosis in cancer patients. However, the use of the coagulation‐related genes (CRGs) for prognosis in LUAD has yet to be determined. In this study, we constructed an immune‐related signature (CRRS) and identified a potential coagulation‐related biomarker (P2RX1).

**Methods:**

We obtained a total of 209 CRGs based on two coagulation‐related KEGG pathways, then developed the CRRS signature by using the TCGA‐LUAD RNA‐seq data via the procedure of LASSO‐Cox regression, stepwise‐Cox regression, univariate and multivariate Cox regression. Grouped by the CRRS, Kaplan–Meier survival curves and receiver operating characteristic curves were drawn for the training and validation sets, respectively. In addition, single‐sample gene set enrichment analysis was exploited to explore immune infiltration level. Moreover, immunophenotypes and immunotherapy grouped by CRRS were further analyzed.

**Results:**

We developed an immune‐related signature (CRRS) composed of COL1A2, F2, PLAUR, C4BPA, and P2RX1 in LUAD. CRRS was an independent risk factor for overall survival and displayed stable and powerful performance. Additionally, CRRS possessed distinctly superior accuracy than traditional clinical variables and molecular features. Functional analysis indicated that the differentially high expressed genes in the low‐risk group significantly enriched in T cell and B cell receptor signaling pathways. The low‐risk group was sensitive to anti‐PD‐1/PD‐L1 immunotherapy and displayed abundant immune infiltration and immune checkpoint gene expression. Finally, we identified an independent prognostic gene P2RX1. Low expression of P2RX1 associated with poor overall survival and decreased immune infiltration.

**Conclusions:**

Our study revealed a significant correlation between CRRS and immune infiltration. CRRS could serve as a promising tool to improve the clinical outcomes for individual LUAD patients.

## Graphical abstract

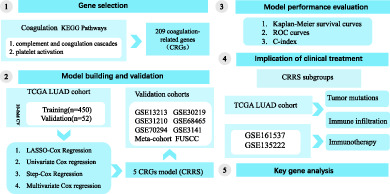

## INTRODUCTION

Lung cancer is the most common tumor with the second highest rate of incidence and mortality worldwide.^1^ Lung adenocarcinoma (LUAD) is the most prevalent pathological subtype of lung cancer, accounting for approximately 40% of all cases,^2^ which results in more than one million related deaths each year. Despite surgery, chemoradiotherapy, targeted therapy, and immunotherapy being used in the treatment of lung cancer, the prognosis remains disheartening with a 5‐year survival of only 4%–17%.^3^, ^4^ Although many biomarkers have been identified as potential predictors of LUAD prognosis, most of them are still in the molecular research phase and have not yet been applied in clinical practice. Therefore, there is an urgent need to identify novel and effective prognostic biomarkers for predicting LUAD prognosis and to carry out personalized therapeutic strategies for patients.

The coagulation system is an innate defense mechanism that can be activated either by the extrinsic or intrinsic pathways.^5^ Cancer patients often have many coagulation abnormalities, which increase the risk of thrombosis and bleeding. Many studies have found that tumor cells can express procoagulant factors, such as tissue factor, which trigger the coagulation cascades leading to thrombin production.^6^, ^7^, ^8^ The activation of coagulation and fibrinolysis interacts directly with malignancy and promotes tumor cell invasion, progression, induction of angiogenesis, and ultimately poor prognosis.^9^ Blockade of coagulation, fibrinolysis, and platelet activation pathways can effectively prevent tumor progression.^10^ Many biomarkers related to coagulation disorders have been confirmed to be significantly related to prognosis in lung cancers.^11^ The impact of coagulation on tumors becomes an area of intense research interest. However, the use of the coagulation‐related genes (CRGs) for prognosis in LUAD has yet to be determined.

Recently, some studies revealed that coagulation can interact with the tumor immune microenvironment (TME) to orchestrate either tumor progression or inhibition and even influence the tumor immune response.^12^, ^13^ In addition, a retrospective clinical study showed that rivaroxaban, a coagulation factor‐targeted drug, can increase the efficacy of immune checkpoint inhibitors (ICIs) by restoring host antitumor immunity.^14^ These findings emphasized the vital roles of coagulation in the TME and tumor immune evasion. TME also plays a crucial role in the development of lung cancer and immunotherapeutic strategies are considered a promising direction for the treatment of lung cancer.^15^ Therefore, we employed bioinformatic approaches to assess the relevance of coagulation with the TME of lung cancer.

To achieve this, we attempted to apply CRGs to develop and validate a risk stratification signature in 2126 LUAD patients from seven independent public datasets and a clinical in‐house cohort to assess the prognosis, recurrence, and benefits of PD‐1/PD‐L1 and ICI treatment in LUAD. Additionally, we identified an independent prognostic predictor P2RX1. This study may help optimize precision treatment and further improve the clinical outcomes of LUAD patients.

## METHODS

### Dataset and preprocessing

**FIGURE 1:**
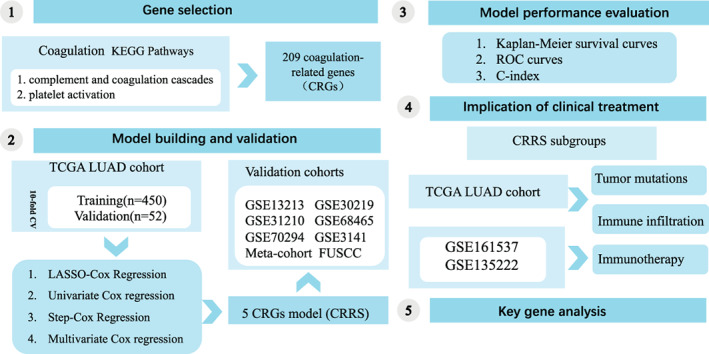
The overall design of this study.

In total, 2027 LUAD patients from seven independent public datasets (TCGA‐LUAD, GSE13213, GSE31210, GSE72094, GSE68465, GSE3141, and GSE30219) were accessed from The Cancer Genome Atlas (TCGA) and Gene Expression Omnibus (GEO). In‐house LUAD cohort (*n* = 99) was collected from Fudan University Shanghai Cancer Center (FUSCC).^16^ These datasets encompassing complete OS information were used for the construction and validation of our signature. For drug‐related datasets, we enrolled two non‐small cell lung cancer (NSCLC) datasets treated with anti‐PD‐1/PD‐L1 (GSE161537 and GSE135222). These drug‐related datasets were applied to assess the performance of CRRS in predicting immunotherapy benefits in LUAD (Figure 1).

RNA‐seq raw read count of these datasets were log2(exp + 1) transformed and normalized before further analysis. The detailed baselines of the seven enrolled datasets are summarized in Supporting Information S1.

### Somatic mutation and copy number variation analysis

A total of 209 CRGs in two coagulation pathways, hsa04610 (complement and coagulation cascades) and hsa04611 (platelet activation)^17^ were downloaded using the R package KEGGREST. It ißs well known that DNA alterations include mutations (truncations and missenses) and copy number variations (amplifications and deep deletions). To reduce the false positive rate, we only kept nonsilent mutations such as missense mutation, nonsense mutation, nonstop mutation, frame shift del, frame shift ins, in frame del, and in frame ins. Mutation frequencies and the OncoPrint plot of 209 CRGs in TCGA LUAD patients were analyzed by the R package “maftools”.^18^ In the copy number variation dataset, values equal to 2 and − 2 were considered being amplifications and deep deletions. We downloaded the somatic copy number alteration (SCNA) stack bar plot from the cBioPortal online tool (https://www.cbioportal.org/).^19^ We analyzed the overall survival (OS) and recurrence‐free survival (RFS) in patients with and without DNA alterations (SCNA and mutation), and the gene expression between with and without SCNA/mutations.

### Construction of a coagulation‐related risk score (CRRS) signature

The signature generation procedure was as follows: (a) LASSO‐Cox regression was performed 100 times on the 209 CRGs to select six prognostic biomarkers based on the 10‐fold cross‐validation framework in the TCGA LUAD cohort; (b) Then, stepwise‐Cox regression identified five prognostic biomarkers; (c) Finally, univariate and multivariate Cox regression generated the optimal signature CRRS; (d) The signature CRRS was validated in eight validation datasets (GSE13213, GSE31210, GSE72094, GSE68465, GSE3141, GSE30219, meta cohort, and FUSCC cohort); (d) Finally, the Harrell's concordance index (C‐index) and receiver operating characteristic (ROC) curve was calculated across all validation datasets.

### Immune cell infiltration estimation

In the TCGA‐LUAD cohort, the gene set variation analysis (GSVA) method of the R package GSVA was used to calculate the normalized enrichment score (NES) of the pathway and functional annotations.^20^ We implemented DEGs‐based gene set enrichment analysis (GSEA) by the R package clusterProfiler, and visualized the GSEA result by the R package GSEA.^21^ The stromal score, immune score, and ESTIMATE score were calculated by the R package estimate.^22^ Single‐sample gene set enrichment analysis (ssGSEA)^23^ implemented in R package GSVA was employed to quantify the relative infiltration of 28 immune cells in the TCGA‐LUAD cohort.

### Differential expression profiling of coagulation‐related genes

Using fold‐change ≥1.2 and FDR <0.05, we performed the differential expression profiling on TCGA and FUSCC LUAD cohort between the tumor and normal tissues.^24^ We performed overlapping analysis on five CRGs and the TCGA and FUSCC DEGs, leading to a key CRG P2RX1.The tumor mutational burden (TMB) was calculated based on the DNA mutation dataset by the R package maftools.

### Statistical analysis

All data processing, statistical analysis, and plotting were conducted in R 4.2.1 software. Kaplan–Meier survival curves were constructed using the ggsurvplot package. The continuous variables were compared through the Wilcoxon rank‐sum test. The survminer package was used to determine the optimal cutoff value. Cox regression and Kaplan Meier analyses were performed via the survival package. The C‐index of different variables were compared using the CompareC package. The ROC curve used to predict binary categorical variables was implemented via the pROC package. The time‐dependent area under the ROC curve (AUC) for survival variables was conducted by the timeROC package. All statistical tests were two‐sided. *p* < 0.05 was regarded as statistically significant.

## RESULTS

### Identification of mutations and copy number variations of coagulation‐related genes

**FIGURE 2:**
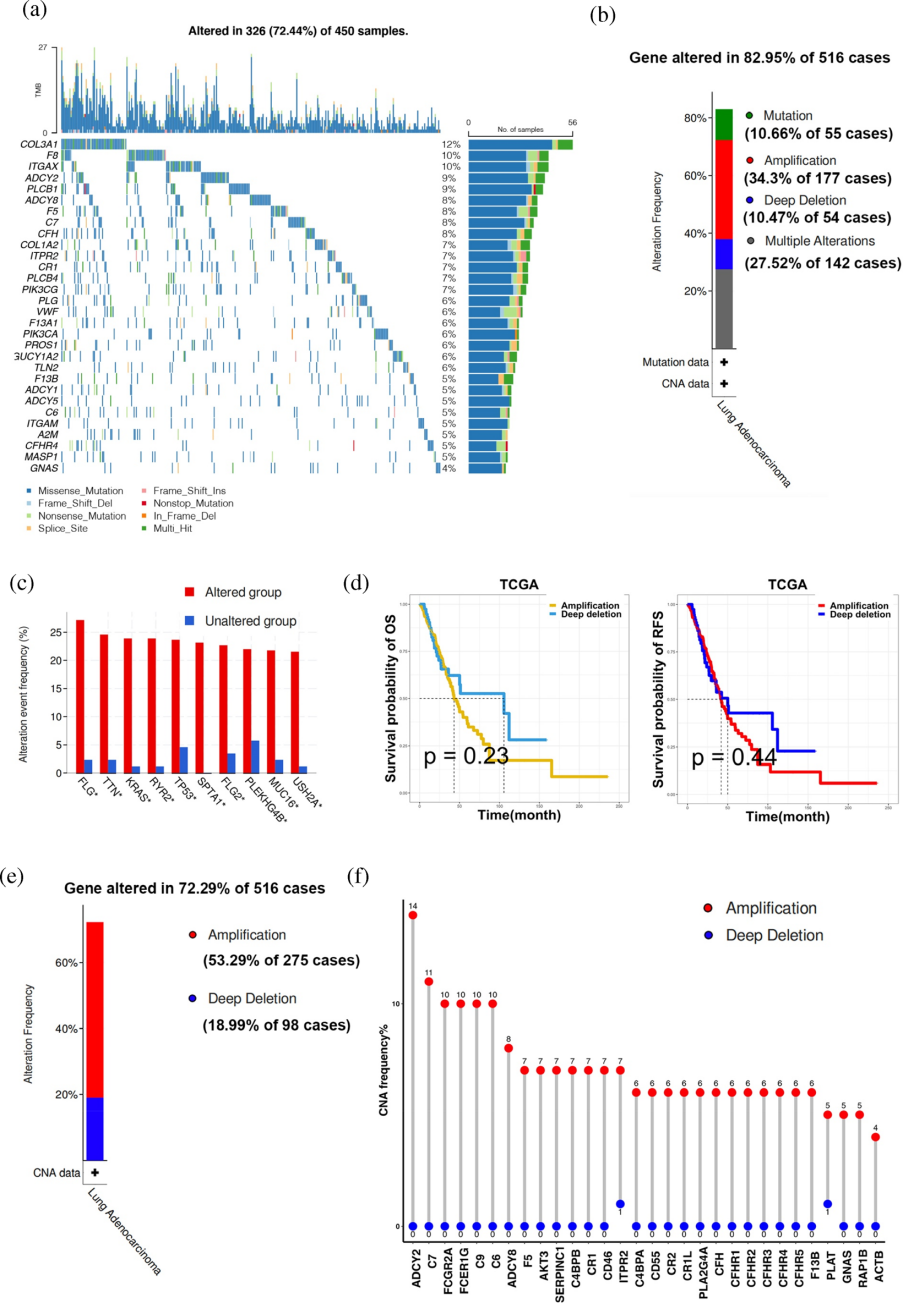
The SCNA of CRGs in lung adenocarcinoma (LUAD). (a) Landscape of the top 30 CRG mutations in LUAD. Each row represented a gene and each column represented a patient. (b) The frequency of alterations in TCGA LUAD patients.^22^ (c) The histogram of different mutational modes in LUAD.^22^ (d) The OS and RFS of LUAD patients between amplification and deep deletion groups. (e) Histogram of the proportion of different CNA types in LUAD.^22^ (f) Lollipop chart of the CNA proportion in CRGs.

For studying the genomic landscape of the CRGs in LUAD, we analyzed somatic copy number variation (SCNA) and mutations in 450 TCGA LUAD patients who had at least one CRG. DNA mutations in the CRGs presented in about 72.44% of patients, and the frequency of DNA mutations in the CRGs ranged from about 4% to 12% (Figure 2a,b). The CRGs with the highest mutation frequency were COL3A1 (12%), F8 (10%), ITGAX (10%), ADCY2 (9%), PLCB1 (9%) and ADCY8 (8%). The main mutation types were missense mutation, frame shift del, nonstop, splice (Figure 2a and Figure S1a). Although we did not observe high mutation frequencies in the CRGs, SCNA accounted for the majority of DNA alterations. About 72.29% of LUAD patients had at least one CRG SCNA (Figure 2e). Among the CRGs, ADCY2, C7, FCGR2A, FCER1G, C9 and C6 had high amplification frequencies (10%–14%) and few deep deletions. Therefore, most CRGs with high CNA frequencies tended to be co‐amplification rather than codeletion (Figure 2f). CNA patients had a higher proportion of multiple tumor suppressor genes, such as TP53, PKHD1L1, and CTNNB1 (Figure 2c). In addition, Kaplan–Meier survival curves showed that patients with low CNA frequency had longer OS and RFS than those with high CNA frequency (Figure 2d,f), but there was no statistically significant difference in prognosis between the high and low mutation groups (Figure S1b). Gene amplification and deletion can change gene expression. We found patients with CNA amplification of some CRGs had higher gene expression than those without alterations, but mutations did not affect gene expression significantly (Figure S1c,d). Taken together, these results suggested SCNA was responsible for dysregulation of CRGs in LUAD rather than mutation.

### Construction of coagulation‐related prognostic signature in LUAD

**FIGURE 3:**
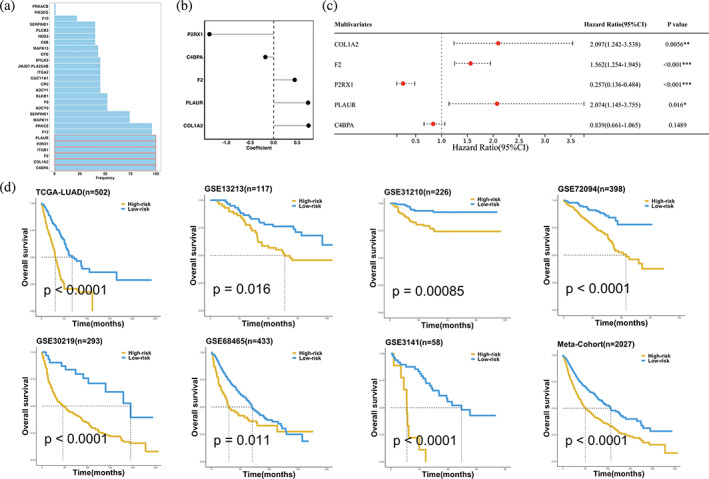
A CRRS model was developed and validated via LASSO‐Cox and stepwise‐Cox procedure. (a) Frequency of selected features via the 100‐times LASSO‐Cox procedure. (b) Coefficients of the five CRGs in the multivariate Cox regression model. (c) Multivariate Cox regression of 5 CRGs regarding to OS in TCGA‐LUAD (*n* = 502). Statistical tests: two‐sided Wald test. Data are presented as hazard ratio (HR) ± 95% confidence interval [CI]. (d) Kaplan–Meier curves of OS according to the CRRS in TCGA‐LUAD (*p* < 0.0001), GSE13213 (*p* = 0.016), GSE31210 (*p* = 0.00085), GSE70294 (*p* < 0.0001), GSE30219 (*p* < 0.0001), GSE68465 (*p* = 0.011), GSE3141 (*p* < 0.0001), and meta‐cohort (*p* < 0.0001).

To determine the correlation between CRGs and TME, we constructed the CRRS based on the TCGA LUAD cohort. The top six CRGs whose frequencies were 100% by 100 times LASSO‐Cox regression analysis were selected (Figure 3a). Then five CRGs were further identified by stepwise‐Cox regression and univariate Cox regression analysis. Next, a risk score for each patient was calculated using the expression of five CRGs weighted by their regression coefficients in a multivariate Cox regression model (Figure 3b). The associations between the expression levels of five CRGs and OS are shown in Figure 3c. The expression levels of COL1A2, F2, and PLAUR had significant positive contributions to better prognosis, while the expression levels of P2RX1 and C4BPA played opposite roles.

All patients were assigned into high‐ and low‐risk groups according to the optimal cutoff value determined by the survminer package. As illustrated in Figure 3d, patients in the high‐risk group had significantly dismal overall survival (OS) relative to the low‐risk group in the TCGA‐LUAD training dataset and six validation datasets (all *p* < 0.05). The meta‐cohort that combined all samples also showed the same trend (*p* < 0.05). Multivariate Cox regression demonstrated that CRRS remained statistically significant in TCGA and FUSCC LUAD cohorts (*p* < 0.05) after adjusting for available clinical traits, such as age; gender; T, N, M, and stage; TMB; MSI; TP53 and EGFR mutations, which suggested that CRRS is an independent risk factor for OS (Figure S2). However, multivariate Cox regression indicated that CRRS remained statistically significant for RFS in TCGA cohort but FUSCC cohort (Figure S2). Hence, for RFS, CRRS had a certain degree of predictive value, but it was not an independent prognostic factor.

### Evaluation of the CRRS model

**FIGURE 4:**
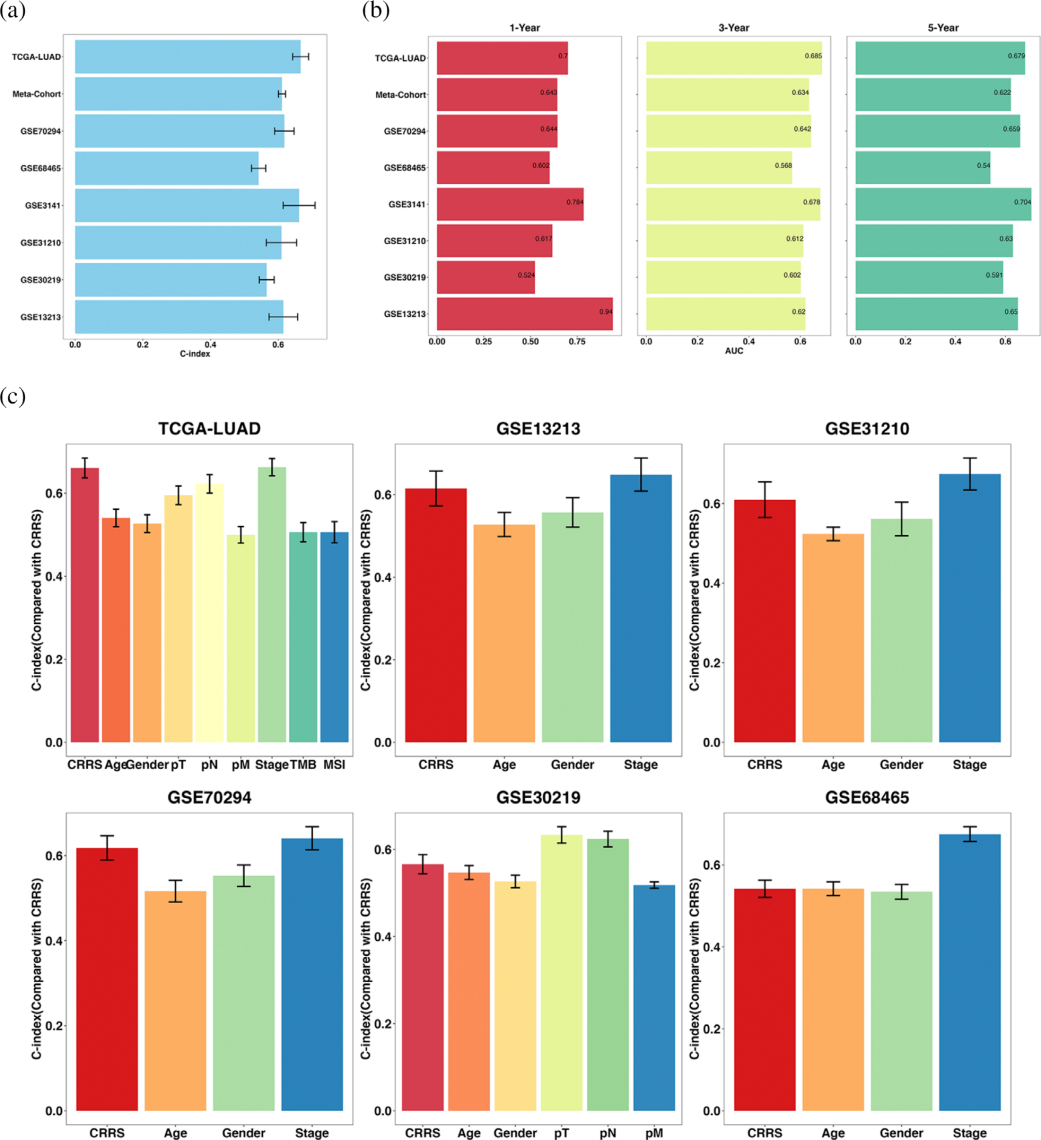
Evaluation of the CRRS model. (a) C‐index of CRRS across all datasets. (b) Time‐dependent ROC analysis for predicting OS at 1, 3, and 5 years. (c) The performance of CRRS was compared with other clinical and molecular variables in predicting prognosis. Data are presented as mean ± 95% confidence interval [CI].

The C‐index was 0.666, 0.618, 0.542, 0.662, 0.610, 0.566, 0.615, and 0.611 in the eight cohorts (TCGA‐LUAD, GSE70294, GSE68465, GSE3141, GSE31210, GSE30219, GSE13213, and meta‐cohort) (Figure 4a). ROC analysis measured the discrimination of CRRS, with 1‐, 3‐, and 5‐year AUCs of 0.700, 0.685, and 0.679 in TCGA‐LUAD; 0.644, 0.642, and 0.659 in GSE70294; 0.602, 0.568, and 0.540 in GSE68465; 0.784, 0.678, and 0.704 in GSE3141; 0.617, 0.612, and 0.630 in GSE31210; 0.524, 0.602, and 0.591 in GSE30219; 0.940, 0.620, and 0.679 in GSE13213; and 0.643, 0.634, and 0.622 in meta‐cohort, respectively (Figure 4b). All these indicators suggested that CRRS had stable and robust performance in multiple independent cohorts. A previous study reported that clinical characteristics (e.g., stage) and molecular alterations (e.g., MSI) were also used to assess the prognosis of LUAD in clinical practice. Therefore, we compared the performance of CRRS with other clinical and molecular variables in predicting prognosis. As displayed in Figure 4c, CRRS had distinctly superior accuracy than the other variables including age; gender; TMB; MSI, excluding stage. An interesting idea is to combine CRRS with commonly used clinical traits to further elevate clinical utility. Stage is a commonly used tool for the clinical management of LUAD, and multivariate Cox regression analysis of stage was statistically significant across multiple cohorts. Thus, we further explored the performance of CRRS + Stage. As shown in Figure S3, we found that the performance of CRRS + Stage was significantly better than that of CRRS or stage alone in multiple datasets. These results led us to conclude that the combination of CRRS and stage may further improve the predictive ability of our model.

### Comparison of coagulation‐related prognostic signatures in LUAD

To compare the performance of CRRS with other coagulation‐related signatures, we comprehensively retrieved published signatures, including Jin's model with 11 signatures (SERPINA1, CFHR3, PPP1CB, P2RX1, PLCB3, PLCB4, PIK3R6, GP6, PIK3R1, GP1BA, PLA2G4F) in HCC,^17^ Chen's model with seven signatures (SERPINE1, VWF, F2R, ANXA5, CD59, AXL, MMRN1) in gastric cancer,^25^ Jia's model with four signatures (SERPINA1, HMGCS2, MMP7, PLAT) in invasive ductal cancer,^26^ Song's model with seven signatures (ANG, C1QA, CFB, DUSP6, KLKB1, MMP7, RABIF) in melanoma.^5^ The AUC mean values for Jin's model, Chen's model, Jia's model, Song's model and the CRRS were 0.641, 0.655, 0.569, 0.486 and 0.696. Furthermore, the C‐index of CRRS was compared with other signatures; notably, CRRS displayed better performance (Figure S5a,b). Furthermore, the p‐value of our CRRS model was the most significant compare to other four models in Kaplan–Meier survival curves (Figure S5c,d). Our CRRS signature's remarkable predictive capabilities were highlighted by such outcomes.

### Validation in FUSCC LUAD cohort

**FIGURE 5:**
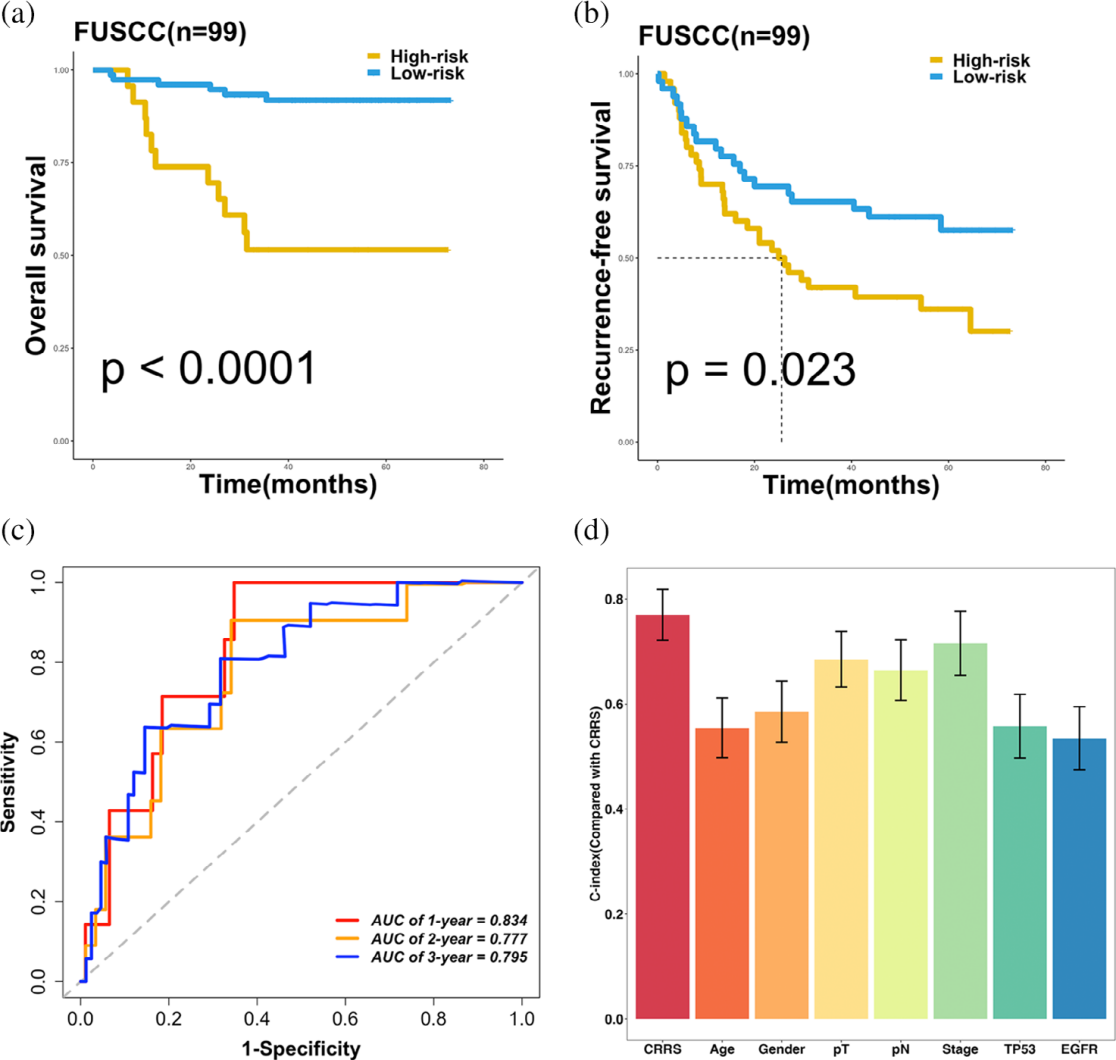
Validation in FUSCC cohort. (a, b) Kaplan–Meier curves of OS (*p* < 0.0001) (a) and RFS (*p* = 0.023). (c) Time‐dependent ROC analysis for predicting OS at 1, 2, and 3 years. (d) The performance of CRRS was compared with other clinical and molecular variables in predicting prognosis in our FUSCC cohort (*n* = 99). Data are presented as mean ± 95% CI.

To further verify the performance of our CRRS model in a clinically translatable tool, we next evaluated the expression of these CRGs in FUSCC LUAD cohort (*n* = 99). Consistently, Kaplan–Meier analysis demonstrated that patients with high CRRS exhibited dramatically worse OS (*p* < 0.0001) and RFS (*p* < 0.023) (Figure 5a,b). After controlling for confounding variables (including age, gender, T, N, stage, TP53 and EGFR mutations), the CRRS model remained statistically significant for OS instead of RFS (Figure S2c,d). ROC analysis showed a superior accuracy of CRRS: the AUCs for predicting OS at 1, 2, and 3 years were 0.834, 0.777, and 0.795, respectively (Figure 5c). Similarly, the C‐index reached 0.770 (95% CI = 0.721–0.819). In addition, we compared the predictive superiority of CRRS with other clinical features and observed that CRRS maintained optimal performance (Figure 5d). Collectively, the results from the FUSCC cohort supported our discovery which validated and confirmed that our CRRS model was quite robust and can serve as an independent predictor of prognosis in LUAD.

### Immune landscape of the CRRS subgroups

**FIGURE 6:**
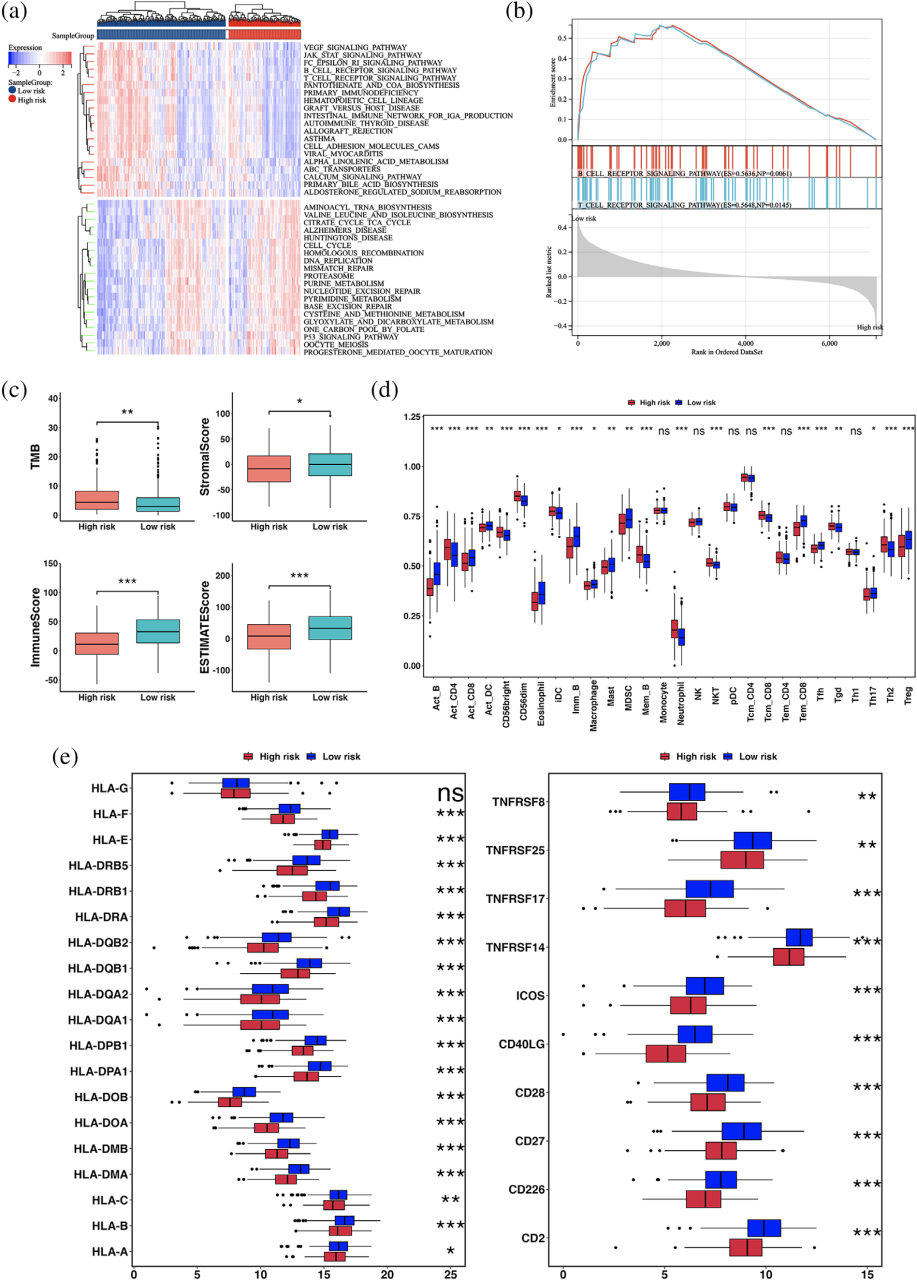
Immunoassay of the CRRS subgroups in the TCGA LUAD cohort. (a) GSVA analysis of the biological pathways between high‐ and low‐CRRS subgroups. Red represented activation of biological pathways and blue represented inhibition of biological pathways. (b) Significant enrichment immune‐related biological pathways by gene set enrichment analysis (GSEA). (c) The TMB, stromal score, immune score, and ESTIMATE score between the two subgroups. (d) Immune cell infiltration between the two subgroups. (e) Gene expression of MHC and T cell stimulation gene sets between the two subgroups. Statistical significance at the level of ns ≥0.05, * < 0.05, ** < 0.01, *** < 0.001 and **** < 0.0001.

To determine difference in pathway enrichment analysis between the high‐ and low‐CRRS subgroups in the TCGA LUAD cohort, GSVA analysis was performed and revealed the two subgroups had distinct immune infiltration patterns. The enrichment heatmap illustrated the low‐CRRS subgroup was significantly enriched in immune and inflammatory pathways, including T cell receptor signaling and B cell receptor signaling (Figure 6a). GSEA analysis confirmed the difference of immune pathways between the two subgroups, and the DEGs with high expression significantly enriched in the T cell and the B cell receptor signaling pathways were observed in the low‐CRRS subgroup (Figure 6b).

To further explore the difference of the immune infiltration between the two subgroups, we calculated TMB, stromal score, immune score, and ESTIMATE score based on the expression profile of TCGA LUAD cohort. The stromal score generated by ESTIMATE algorithm, which captured the presence of stroma in the tumor tissue, and the Immune score which represented the infiltration of the immune cell in the tumor tissue, and ESTIMATE score inferred tumor purity. As shown in Figure 6c, compared with the high‐CRRS subgroup, higher stromal score, immune score, ESTIMATE score, and lower TMB were observed in the low‐CRRS subgroup (Wilcoxon test, *p* < 0.05). The low‐CRRS subgroup was characterized by higher infiltration of activated B cell (Act_B), activated CD8+ T cell (Act_CD8), activated dendritic cell (Act_DC), eosinophil, immature B cell (Imm_B), macrophage, mast cell (Mast), MDSC, effector memory CD8+ T cell (Tem_CD8), follicular helper T cell (Tfh), type 17 T helper cell (Th17), and regulatory T cell (Treg), and high‐CRRS subgroup was characterized by higher infiltration of activated CD4+ T cell (Act_CD4), CD56bright natural killer cell (CD56bright), CD56dim natural killer cell (CD56dim), immature dendritic cell (iDC), memory B cell (Mem_B), neutrophils, natural killer T cell (NKT), central memory CD8+ T cell (Tcm_CD8), gamma delta T cell (Tgd), and type 2 T helper cell (Th2) (Figure 6d). Then, we analyzed the differences in major histocompatibility complex (MHC) and T cell stimulating factors between the two subgroups. Excluding HLA‐G, higher expression of MHC and T cell stimulating factors were observed in the low‐CRRS subgroup (Figure 6e,f). These findings indicated the high‐ and low‐CRRS subgroups had different patterns of tumor immune infiltration, which indicated that the CRRS significantly correlated with the TME and may be sensitive to the response to immunotherapy.

### CRRS‐based LUAD immunotherapy strategy

Currently, one of the bottlenecks in developing immunotherapy was the absence of new predictive biomarkers. Since the immune infiltration patterns in high‐ and low‐CRRS subgroups were proved significantly different, we further analyzed whether CRRS could predict the response to immunotherapy.

**FIGURE 7:**
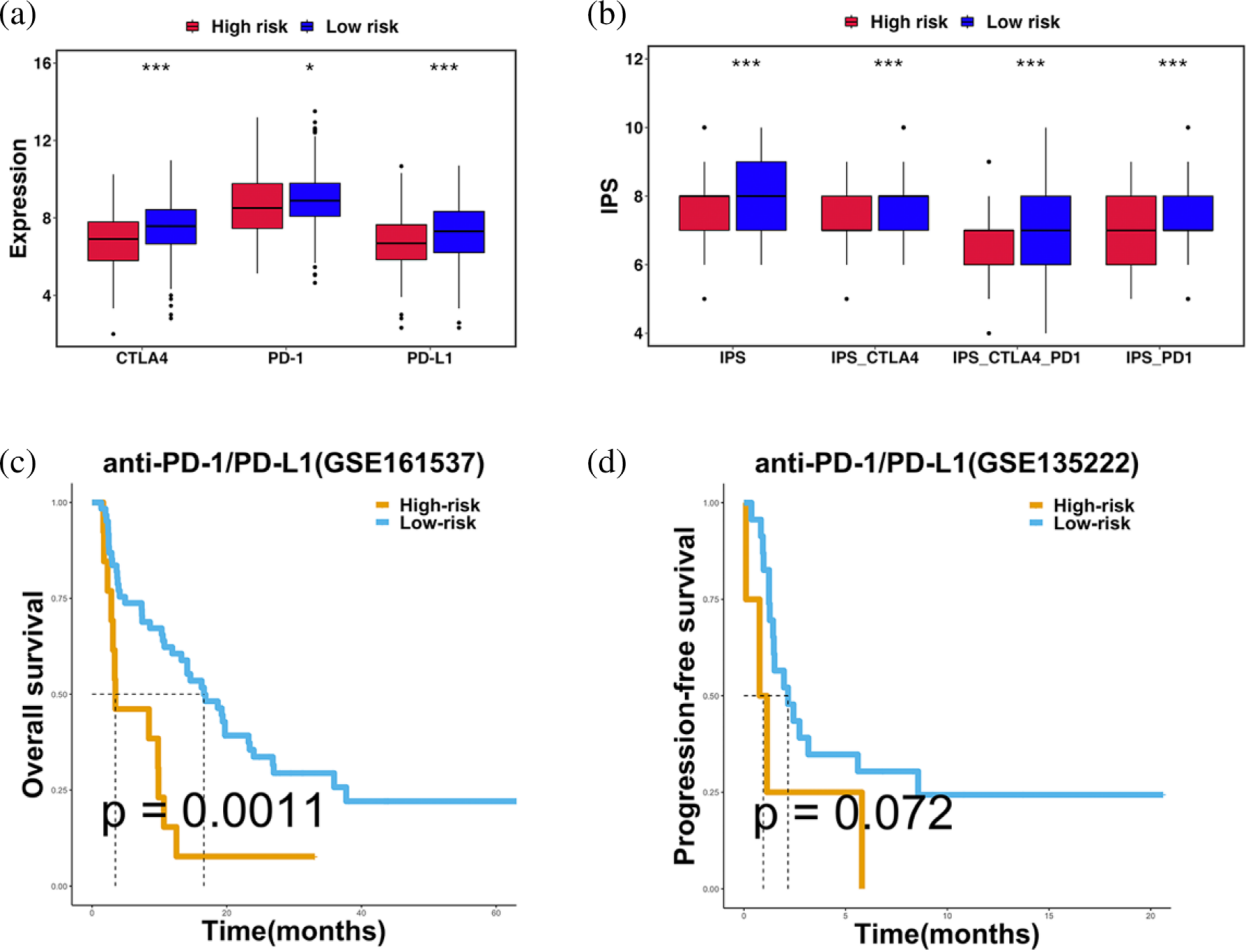
The evaluation of the CRRS for predicting response to immunotherapy. (a) The expression of three immune checkpoint molecules (CTLA4, PD‐1, PD‐L1) in high‐ and low‐CRRS subgroups. (b) IPS, IPS‐CTLA4, IPS‐PD1‐CTLA44, and IPS‐PD1 scores between the two CRRS subgroups. (c) Kaplan–Meier curves of OS in GSE161537 (*p* = 0.0011). (d) Kaplan–Meier curves of OS in GSE135222 (*p* = 0.072). Statistical significance at the level of ns ≥0.05, * < 0.05, ** < 0.01, *** < 0.001 and **** < 0.0001.

To assess the ability of CRRS as a biomarker for predicting the response to ICIs, we compared the expression of three immune checkpoint molecules (PD1, PD‐L1, and CTLA4) in high‐ and low‐CRRS subgroups. Four immune checkpoint molecules (PD1, PD‐L1, and CTLA4) were significantly higher in the low‐CRRS subgroup (Figure 7a). IPS, IPS‐CTLA4, IPS‐PD1‐CTLA4, IPS‐PD1 immunophenotype scores were quantitative indicators for evaluating the effectiveness of ICIs. In the low‐CRRS subgroup, IPS, IPS‐CTLA4, IPS‐PD1‐CTLA4, IPS‐PD1 scores were significantly higher (Figure 7b). Kaplan–Meier curves of OS in two NSCLC anti‐PD‐1/PD‐L1 cohorts (GSE161537: *p* = 0.0011; GSE135222: *p* = 0.072) showed that the low‐risk subgroup had a better prognosis which indicated that the low‐risk subgroup may benefit from the anti‐PD‐1/PD‐L1 immunotherapy (Figure 7c,d).

### Identification of key coagulation‐related gene

**FIGURE 8:**
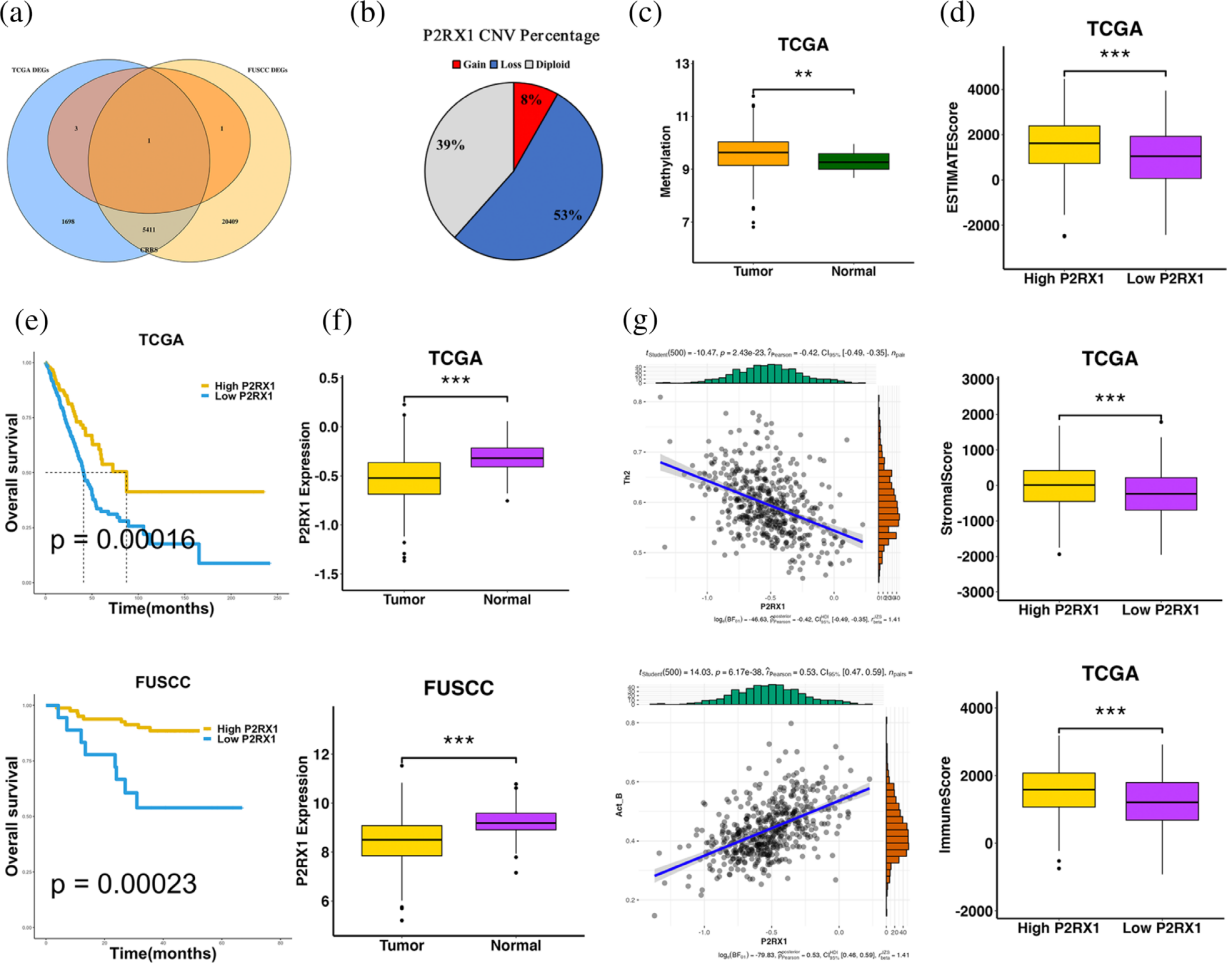
Identification of the key prognostic CRG P2RX1. (a) Venn diagram of overlapping analysis of five CRRS genes set and DEGs of the TCGA‐LUAD dataset and FUSCC‐LUAD datasets. (b) Pie plot of P2RX1 CNV percentage. (c) The P2RX1 methylation level between TCGA‐LUAD tumor and normal tissues. (d) The ESTIMATE score, stromal score, and immune score grouped by high and low P2RX1 expression. (e) Kaplan–Meier curves of overall survival in TCGA‐LUAD (*p* = 0.00016) and FUSCC (*p* = 0.00023). (f) The P2RX1 expression level between tumor and normal tissues in TCGA‐LUAD and FUSCC LUAD datasets. (g) Scatterplots between Th2 cells (*r* = −0.42, *p* = 2.43e–23) and Act B cells (*r* = 0.53, *p* = 6.17e‐38) with P2RX1 expression are shown in TCGA‐LUAD. Statistical significance at the level of ns ≥0.05, * < 0.05, ** < 0.01, *** < 0.001 and **** < 0.0001.

To identify the key CRGs in CRRS, we performed overlapping analysis of DEGs in TCGA and FUSCC LUAD cohorts, and found a key CRG P2RX1(Figure 8a). In the TCGA LUAD cohort, CNV analysis revealed that P2RX1 had higher proportion of SCNA, with higher proportion of deletion than amplification, and with copy number deletion up to 53% (Figure 8b). Methylation analysis showed the methylation of P2RX1 was significantly higher in tumor than in normal samples (Figure 8c). Transcriptome differential expression analysis showed that P2RX1 was significantly down‐regulated in tumor compared with normal samples (Figure 8f). It is well known that gene expression is negatively correlated with methylation, while the abundance of CNA had a positive effect on gene expression.

To better understand the relevance of P2RX1 expression on the TME, the stromal score, immune score and ESTIMATE score were calculated based on the expression profile of the TCGA‐LUAD cohort. The stromal score, immune score, and ESTIMATE score in the P2RX1 high expression group were significantly higher than those in the P2RX1 low expression group (Figure 8d). The Kaplan–Meier survival curve showed that overexpression of P2RX1 was significantly associated with better OS (*p* = 0.00016) in TCGA‐LUAD cohort and OS (*p* = 0.00023) in FUSCC LUAD cohort (Figure 8e). The expression of P2RX1was negatively correlated with Th2 cells (*r* = −0.42, *p* = 2.43e‐23) and positively correlated with Act B cells (*r* = 0.53, *p* = 6.17e–38) (Figure 8g).

These findings confirmed P2RX1 was a key prognostic protective factor associated with the TME, and copy number amplification and methylation may be important causes for the upregulation of P2RX1 in LUAD.

## DISCUSSION

The incidence and mortality of lung cancer have been consistently increasing year by year.^27^ Despite the continuous improvement in therapeutic drugs and treatment methods, effective treatment of lung cancer in case of recurrence or metastasis has remained challenging.^4^ Previous studies found interactions and effects between malignant tumor progression and coagulation.^28^, ^29^ It has been reported that hyperfibrinogen and hypercoagulability are associated with rapidly growing tumors.^30^ The activation of coagulation and complement and coagulation cascades are correlated with overall survival in lung cancer patients.^31^ Therefore, it is critical to identify coagulation‐related tumor response markers and to develop effective and novel coagulation‐related prognostic models. In this study, we established a prognostic risk model that included 5 CRGs as determined by LASSO‐Cox regression, stepwise‐Cox regression, univariate and multivariate regression analyses. Moreover, the gene signature was found to be highly associated with tumor‐infiltrated immune cells, which might be used as an independent factor. The prognostic risk model consisting of COL1A2, F2, PLAUR, C4BPA, and P2RX1 acts as a novel potential biomarker for evaluating the prognosis and the efficacy of immunotherapy in LUAD.

In this study, we hoped to obtain the coagulation pathway as entire as possible. Therefore, we searched the keywords including platelet and coagulation in the KEGG pathway database. Subsequently, we selected all 209 genes in hsa04610 (complement and coagulation cascades) and hsa04611 (platelet activation) for the next analysis. Based on LASSO Cox regression analysis and univariate Cox regression, we identified 5 prognostic CRGs (COL1A2, F2, PLAUR, C4BPA, and P2RX1). Therein, COL1A2, F2, and PLAUR were the unfavorable genes for the outcome, whereas other genes presented protective function on the prognosis of LUAD patients. Cancer‐associated fibroblasts (CAFs) are one of the most important components of the TME and show a high expression of COL1A2.^32^ F2 is known as coagulation factor. The high expression of F2 is associated with the bad prognosis in LUAD.^33^ The expression of PLAUR correlated with the hallmark gene set “TNFα‐signaling via NFκB”, which has a direct pro‐coagulant effect. Upon exposure to TNFα, cancer cells increase their expression of TF and produce TF‐bearing microparticles with potent local pro‐coagulant effects.^34^, ^35^ P2RX1, a member of the purinergic receptor family, was found to have impact on prognosis and immune cell infiltration in several tumors,^36^, ^37^ and low P2RX1 expression was associated with poor prognosis in LUAD.^38^ It has been confirmed that overexpression of C4BPA was associated with a higher proportion of infiltrating immune cells and better prognosis in patients with LUAD, as well as being correlated with apoptosis.^39^, ^40^ These previous studies have revealed that several genes were closely related to tumor progression of LUAD, which also further confirmed the validity and reliability of our prognostic model.

Nowadays, it has become a growing focus for research to construct prognostic models based on coagulation system to predict the prognostic or diagnostic value in tumors.^5^, ^17^, ^25^, ^26^ However, the power of prognostic prediction of LUAD patients using our signature is better than their signatures. In this study, patients in low‐CRRS group had better prognosis than patients in high‐CRRS group. Univariate and multivariate Cox regression analysis showed that the CRRS signature was an independent prognostic factor.

Tumor immunotherapy stimulates the body's immune function by increasing the immunogenicity of tumor cells and the sensitivity of effector cell killing, thereby inhibiting and killing tumor cells. The coagulation system plays an essential role in innate and adaptive immunity.^41^ Given the importance of tumor immune infiltration, we performed the correlation analysis of immune infiltrates using ssGSEA and stromal score algorithms. Of course, the relationship between immune cells and tumors was extremely complex, and different immune cells had different roles. Recent studies have shown that Th2 cells have tumor‐promoting effects in lung cancer and even human primary NSCLC tumors.^42^, ^43^, ^44^ Neutrophilia has been reported to be a marker of poor prognosis in solid tumors.^45^ The iDCs cells are immunosuppressive cells that lack co‐stimulatory molecules and have a weak ability to present antigens, inhibiting immune activation.^46^ Consistent with the above findings, we identified that higher infiltrations of iDCs, neutrophils, and Th2 which promote tumor immunosuppression were associated with a worse cumulative survival of LUAD patients. Moreover, Existing evidence shows that tumor‐infiltrating B cells play a role in almost all stages of lung cancer.^47^ CD8+ T cells are cytotoxic cells that induce antitumor responses by producing interferon‐(IFN) gamma.^48^ We found that higher infiltrations of B cells and CD8+ T cells which promote tumor immunity were associated with a better cumulative survival of LUAD patients, these findings which were well in line with above research. Furthermore, we found that the low CRRS signature with high MHC and T‐cell stimulating factors was associated with a better prognosis. We also found that CRRS was negatively correlated with immune checkpoint expression and IPS score, which indirectly suggested that CRRS might play a key role in predicting immunotherapy efficacy and patients in the high‐CRRS subgroup may benefit from the immunotherapy. Thus, it is significant to provide and improve the reliability and efficacy of survival risk prediction for LUAD patients by using the novel prognostic signature.

Of course, our study has some limitations that should be acknowledged. First, we demonstrated that five CRGs are associated with prognosis in LUAD, but this was evaluated solely by data mining. More investigations were needed to reveal these 5 genes' functions, and the association between CRGs and the development of LUAD needed to be further explored. Second, while we identified the correlation of coagulation pathways and TME in LUAD patients, the biological mechanisms underlying these phenomena are unknown. Therefore, large‐scale prospective studies about functional and mechanistic experiments were needed to validate and interpret the role of coagulation pathways in LUAD. Thirdly, the median of CRRS was used to divide LUAD patients into high‐ and low‐CRRS subgroups, but the optimal cut‐off value for the CRRS may be a better stratification strategy for LUAD patients. Finally, due to the lack of public datasets of immunotherapy in LUAD, we collected the immunotherapy datasets for melanoma instead. However, it may lead to potential bias for CRRS prediction of immunotherapy in LUAD.

Nonetheless, our study has advantages of investigating the correlation between coagulation and TME of LUAD, focusing on CRGs to assess the prognostic value in LUAD. We also explored the association between the CRRS model and immune infiltrating cells, as well as immune checkpoint expression, which can lead to a new predictive model and therapeutic strategy for the immunotherapy in LUAD. The CRRS is a powerful tool for LUAD survival prediction and guiding clinical treatment, and can help to define the prognosis of LUAD patients and stratify LUAD patients who benefit from antitumor immunotherapy. In conclusion, our systematic study of CRGs provided valuable insights into the role of CRGs in the LUAD TME.

In conclusion, in this study we revealed a significant correlation between 5 CRGs and immune infiltration and confirmed that the CRRS can serve as an independent predictor of LUAD. Although we lacked enough LUAD datasets with immunotherapy, we verified the application value of the CRRS in predict the response of immunotherapy in one NSCLC cohort and four melanoma cohorts. Patients in the high‐CRRS subgroup may benefit from the immunotherapy. We also identified an independent prognostic predictor P2RX1. The CRRS model provided new insights and targets for the diagnosis, prognosis prediction, and treatment management of LUAD patients.

## AUTHOR CONTRIBUTIONS

Haiquan Chen, Ting Ye, Yue‐Qing Hu, and Siqian Yang designed the study. Siqian Yang, Shiqi Chen, and Yue Zhao performed data analysis. Siqian Yang and Tao Wu drafted the manuscript. Siqian Yang, Yuquan Wang, Tingting Li, and Liwan Fu collected datasets. Yue‐Qing Hu helped revise the manuscript. All authors contributed to the article and approved the submitted version.

## CONFLICT OF INTEREST STATEMENT

The authors declare that there is no competing interest.

## Supporting information


**Data S1.** Supplementary Information.

**Figure S1.** The expression of high‐frequency SCNAs between altered and unaltered groups. (a) The mutation types of the top six genes with high frequency mutations. (b) The OS and RFS of patients between CNA and Non‐CNA groups. (c) The OS and RFS of patients between mutation and non‐mutation groups. (d) The expression of the top six genes with high frequency copy number amplification between diploid and Amplification groups. (e) The expression of the top 6 genes with high frequency mutation between mutation and non‐mutation groups.
**Figure S2.** Multivariate Cox regression of CRRS regarding to OS and RFS in TCGA‐LUAD (*n* = 502) and FUSCC (*n* = 99).
**Figure S3.** The performance of CRRS + stage was compared with CRRS and stage alone in predicting prognosis in TCGA‐LUAD, GSE13213, GSE31210, GSE70294, GSE30219, and GSE68465.
**Figure S4.** Landscapes of 5 CRRS and top 30 CRGs mutations in TCGA and FUSCC cohorts.
**Figure S5.** Comparison of the CRRS model with published coagulation‐related signatures (a) The time‐ROC curves of the CRRS and other coagulation‐related signatures. (b) The C‐index curves of the CRRS and other coagulation‐related signatures. (c, d) Kaplan–Meier curves of the CRRS and other coagulation‐related signatures.

## Data Availability

The datasets presented in this study can be found in online repositories. The names of the repositories and accession number(s) are TCGA‐LUAD, GSE13213, GSE31210, GSE72094, GSE68465, GSE3141, GSE30219, GSE161537 and GSE135222. The FUSCC datasets presented in this study can be found in online repositories. The names of the repository/repositories and accession number(s) can be found below: European Genome phenome Archive (EGA). Accession number: EGAS00001004006 (“https://ega-archive.org/studies/EGAS00001004006)”. Source data and codes for generating the figures in this study are available at https://github.com/yang-siqian/Coagulation_LUAD_project.
