# Supplementary material for: Identification of a coagulation‐related signature correlated with immune infiltration and their prognostic implications in lung adenocarcinoma
Source: Thorac Cancer. 2023 Oct 5;14(33):3295–308. doi: 10.1111/1759-7714.15121 (PMC10665780; doi:10.1111/1759-7714.15121)
Supplement: Supplementary file 2 — Figure S1. The expression of high‐frequency SCNAs between altered and unaltered groups. (a) The mutation types of the top six genes with high frequency mutations. (b) The OS and RFS of patients between CNA and Non‐CNA groups. (c) The OS and RFS of patients between mutation and non‐mutation groups. (d) The expression of the top six genes with high frequency copy number amplification between diploid and Amplification groups. (e) The expression of the top 6 genes with high frequency mutation between mutation and non‐mutation groups. Figure S2. Multivariate Cox regression of CRRS regarding to OS and RFS in TCGA‐LUAD (n = 502) and FUSCC (n = 99). Figure S3. The performance of CRRS + stage was compared with CRRS and stage alone in predicting prognosis in TCGA‐LUAD, GSE13213, GSE31210, GSE70294, GSE30219, and GSE68465. Figure S4. Landscapes of 5 CRRS and top 30 CRGs mutations in TCGA and FUSCC cohorts. Figure S5. Comparison of the CRRS model with published coagulation‐related signatures (a) The time‐ROC curves of the CRRS and other coagulation‐related signatures. (b) The C‐index curves of the CRRS and other coagulation‐related signatures. (c, d) Kaplan–Meier curves of the CRRS and other coagulation‐related signatures. [file TCA-14-3295-s001.docx]

Supplementary Material

# Supplementary Figures

**
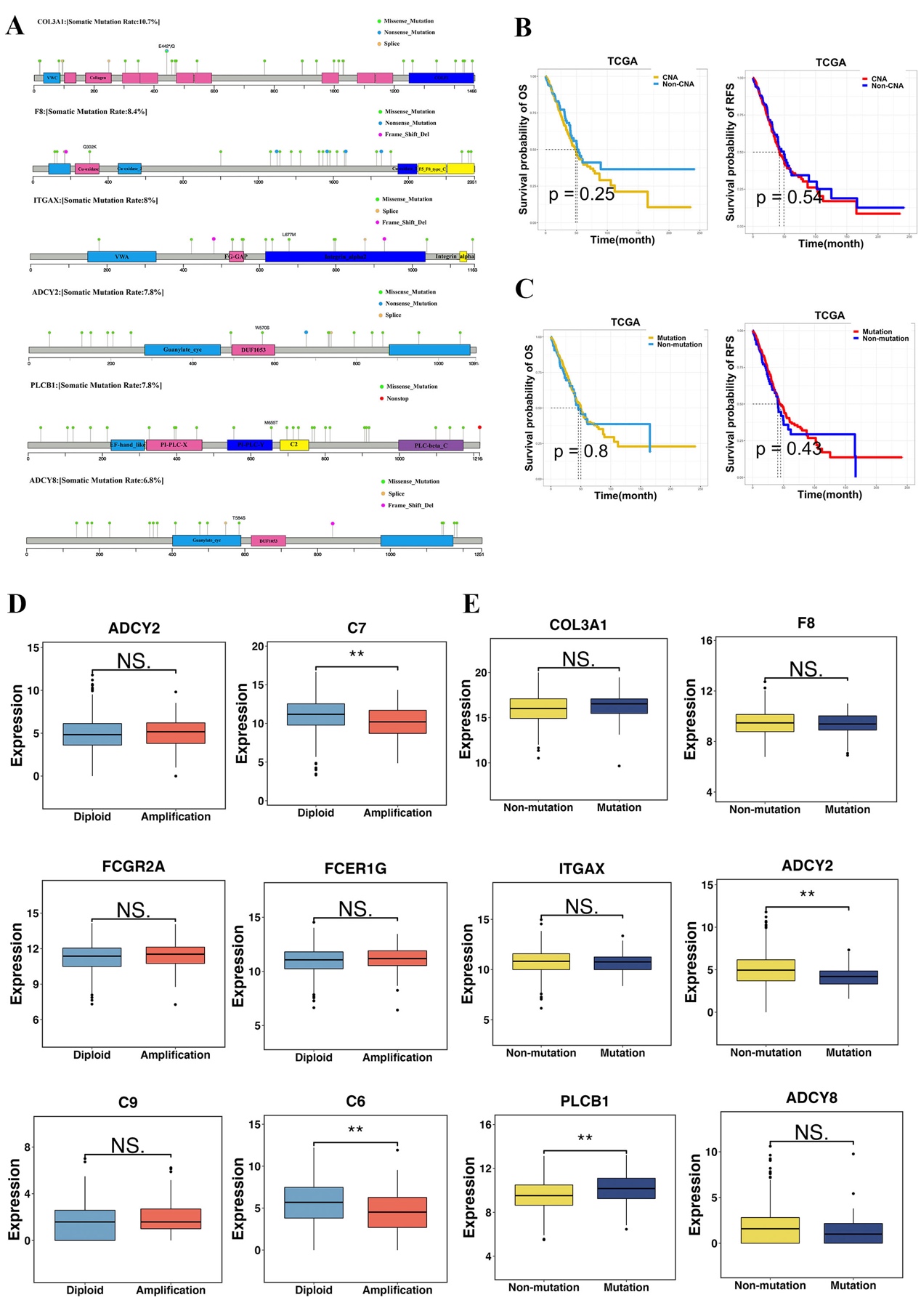
**

**Supplementary Figure 1.** The expression of high-frequency SCNAs between altered and unaltered groups. (A) The mutation types of the top 6 genes with high frequency mutations. (B) The OS and RFS of patients between CNA and Non-CNA groups. (C) The OS and RFS of patients between Mutation and Non-mutation groups. (D) The expression of the top 6 genes with high frequency copy number amplification between Diploid and Amplification groups. (E) The expression of the top 6 genes with high frequency mutation between Mutation and Non-mutation groups.

**
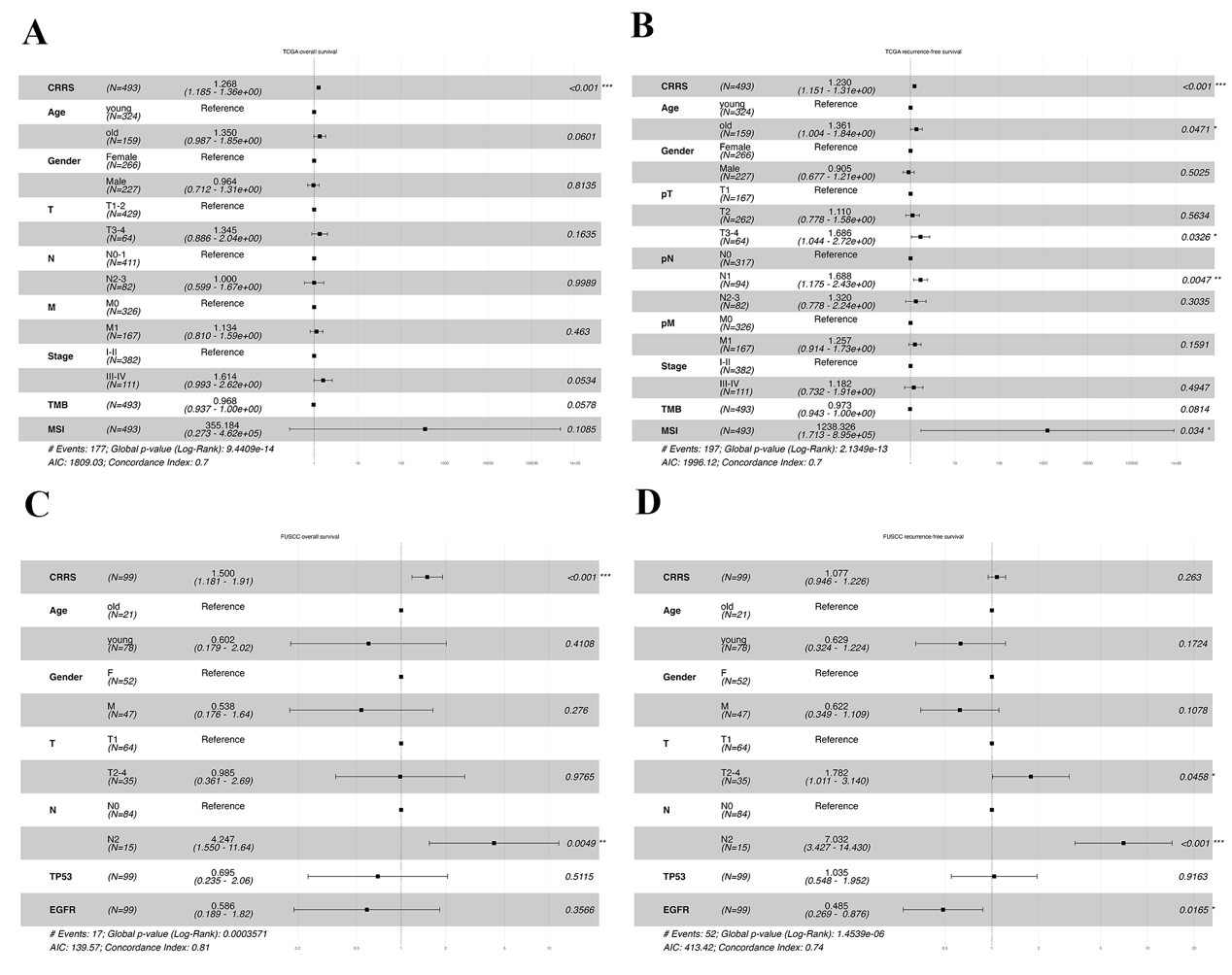
**

**Supplementary Figure 2.** Multivariate Cox regression of CRRS regarding to OS and RFS in TCGA-LUAD (n =502) and FUSCC (n =99).

_­
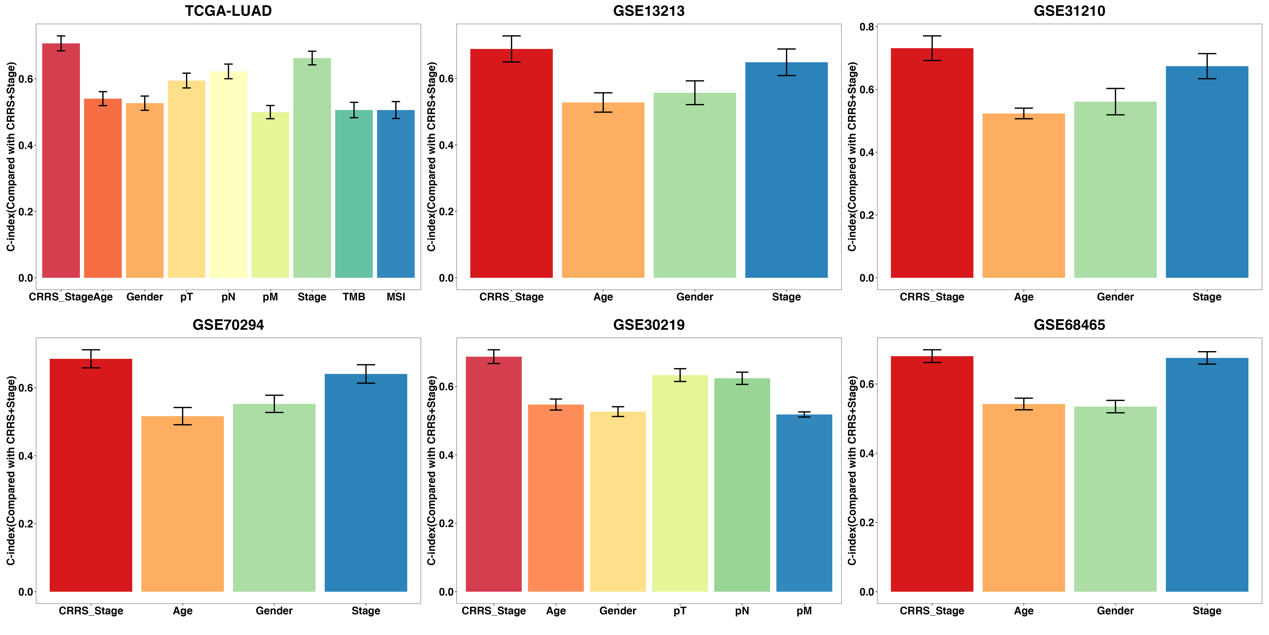
_

**Supplementary Figure 3.** The performance of CRRS+Stage was compared with CRRS and stage alone in predicting prognosis in TCGA-LUAD, GSE13213, GSE31210, GSE70294, GSE30219, and GSE68465.


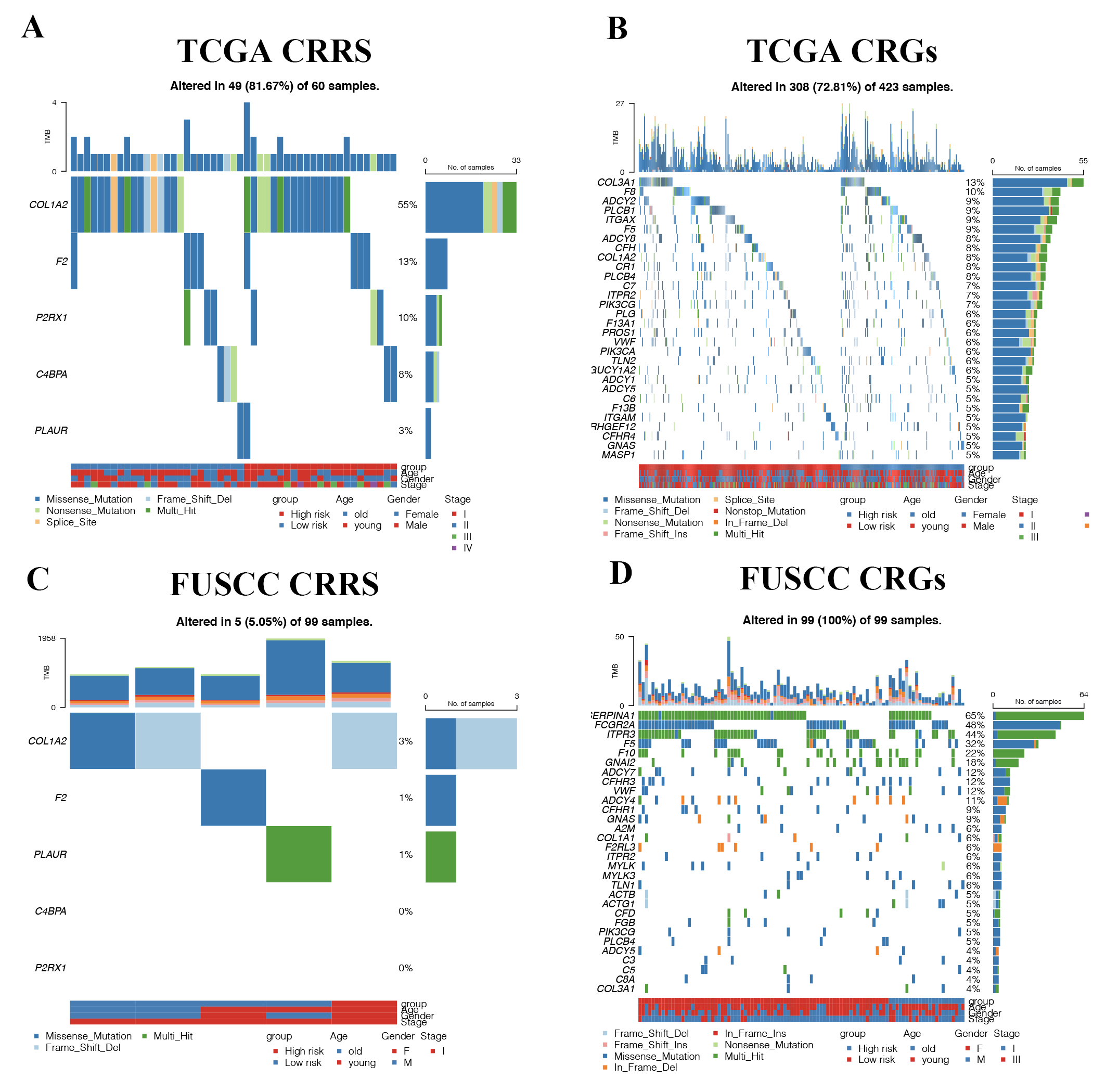


**Supplementary Figure 4.** Landscapes of 5 CRRS and top 30 CRGs mutations in TCGA and FUSCC cohorts.

**
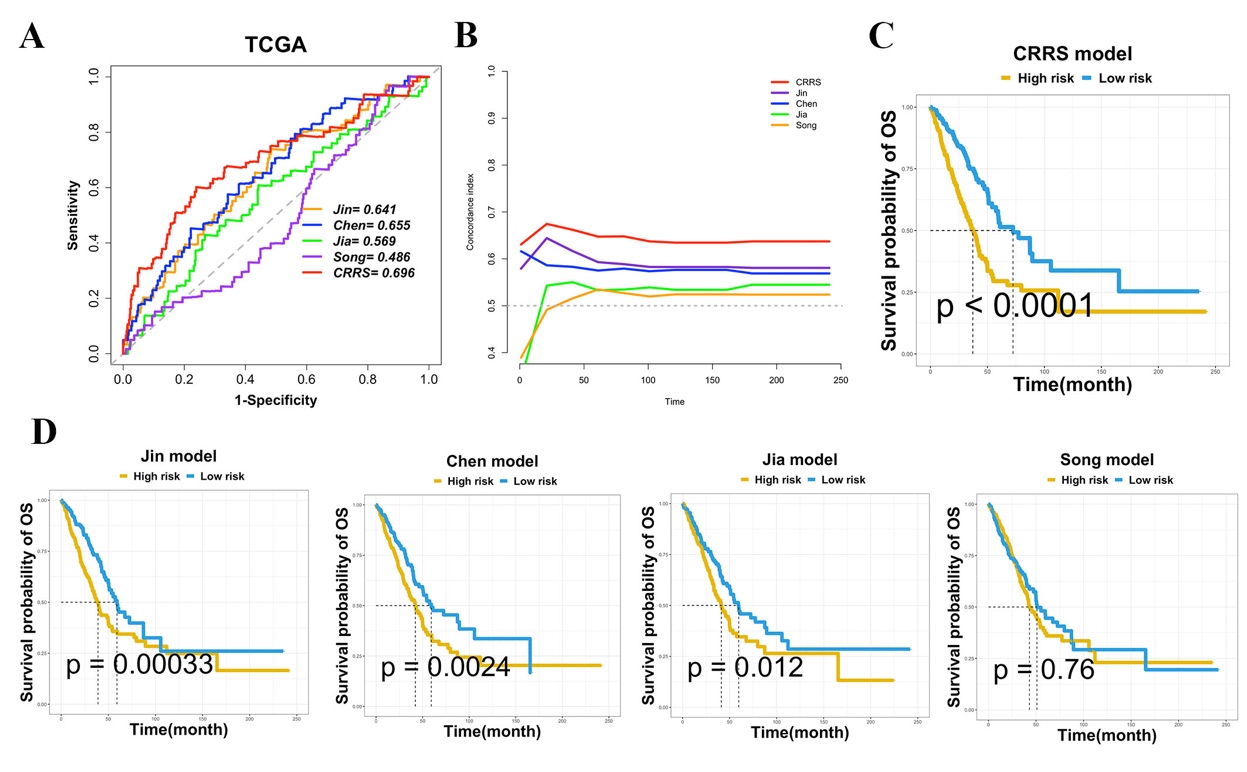
**

**Supplementary Figure 5.** Comparison of the CRRS model with published coagulation-related signatures (A) The time-ROC curves of the CRRS and other coagulation-related signatures. (B) The C-index curves of the CRRS and other coagulation-related signatures. (C-D) Kaplan-Meier curves of the CRRS and other coagulation-related signatures.
